# Fosfomycin-Containing Regimens for the Treatment of Central Nervous System Infections in a Neonatal Intensive Care Unit: A Case Series Study

**DOI:** 10.3390/antibiotics13070667

**Published:** 2024-07-18

**Authors:** Angelica Lenzi, Barbara Saccani, Marco Di Gregorio, Francesco Rossini, Alessio Sollima, Alice Mulè, Federica Morucci, Silvia Amadasi, Benedetta Fumarola, Paola Antonia Lanza, Silvia Lorenzotti, Evelyn Van Hauwermeiren, Elisa Cavalleri, Roberto Marzollo, Alberto Matteelli, Liana Signorini, Francesco Maria Risso

**Affiliations:** 1 Unit of Infectious and Tropical Diseases, Department of Clinical and Experimental Sciences, University of Brescia and ASST Spedali Civili di Brescia, 25123 Brescia, Italy; barbara.saccani@asst-spedalicivili.it (B.S.); m.digregorio002@unibs.it (M.D.G.); f.rossini009@unibs.it (F.R.); a.sollima@unibs.it (A.S.); a.mule@unibs.it (A.M.); f.morucci001@studenti.unibs.it (F.M.); silvia.amadasi@asst-spedalicivili.it (S.A.); benedetta.fumarola@asst-spedalicivili.it (B.F.); paola.lanza@asst-spedalicivili.it (P.A.L.); silvia.lorenzotti@asst-spedalicivili.it (S.L.); efu@hotmail.it (E.V.H.); liana.signorini@asst-spedalicivili.it (L.S.); 2 Neonatology and Neonatal Intensive Care Unit, Children’s Hospital, University of Brescia and ASST Spedali Civili di Brescia, 25123 Brescia, Italy; elisa.cavalleri@asst-spedalicivili.it (E.C.); roberto.marzollo@asst-spedalicivili.it (R.M.); francesco.risso@asst-spedalicivili.it (F.M.R.)

**Keywords:** fosfomycin, central nervous system, CNS infections, meningitis, neonatal intensive care unit, NICU, neonatal meningitis

## Abstract

Central nervous system infections are among the most severe infectious conditions in the neonatal period and are still burdened by significant mortality, especially in preterm infants and those with a low birth weight or other comorbidities. In this study, we examined the role of fosfomycin-containing antibiotic regimens in neonates with central nervous system infections. We included six neonates over a period of five years: four with meningitis and two with cerebral abscesses. All patients underwent fosfomycin therapy after failing first-line antibiotic regimens. Of the six neonates, two died; two developed neurological and psychomotor deficits and two recovered uneventfully. None of the neonates experienced adverse reactions to fosfomycin, confirming the safety of the molecule in this population. In conclusion, the deep penetration in the central nervous system, the unique mechanism of action, the synergy with other antibiotic therapies, and the excellent safety profile all make fosfomycin an attractive drug for the treatment of neonatal central nervous system infections.

## 1. Introduction

Fosfomycin, an old antibiotic developed more than 45 years ago, is showing promising results in the treatment of pediatric infections. Although there is still limited experience with its use, fosfomycin presents an excellent safety profile in children [1], even in prolonged therapies [2].

The indications for intravenous fosfomycin therapy in children are similar to those in adults and include urinary tract infections (UTIs), osteoarticular infections [3], bloodstream infections, and endocarditis. Other infections that can be treated with fosfomycin include nosocomial pneumonia, ventilator-associated pneumonia, and acute gastrointestinal infections, especially in premature neonates [4].

The literature regarding the use of fosfomycin in the treatment of infections of the central nervous system (CNS) is scarce and usually limited to case reports [5].

Fosfomycin has a large volume of distribution (approximately 0.3 L/kg in healthy people) that increases in subjects with sepsis. It has a renal route of elimination; therefore, potentially clinically relevant variability may be observed in critically ill patients who may undergo major changes in membrane permeability and renal function. Furthermore, fosfomycin appears to have a time-dependent or concentration-dependent bactericidal action depending on the pathogen. In the adult population, several studies are available on the pharmacokinetics/pharmacodynamics of fosfomycin, with Monte Carlo simulation conducted for the efficacy of different administration schedules in different pathogens at varying MICs [6]. On the contrary, the pediatric doses of fosfomycin are based on very limited data, especially regarding newborns. Traunmüller et al. conducted a pharmacokinetic study in children which showed that 6–8 h intervals are preferable, although intravenous administration every 12 h is also possible [7]. Darlow et al. recently conducted a study using pharmacokinetic (PBPK) models based on neonatal physiology and concluded that a fosfomycin regimen of 100 mg/kg q12 h for 0–7 days after birth and 150 mg/kg q12 h for 8–28 days after birth has a high likelihood of achieving target levels in both term and preterm newborns [8]. Currently, from a technical data sheet, in preterm newborns, the recommended dose is 100 mg/kg/day divided into two doses, whereas for full-term newborns, an administration of 200 mg/kg/day divided into three doses is recommended.

Bacterial infections of the CNS have significant mortality rates in the neonatal period [9]. The most common pathogens that are implicated in neonatal meningitis are *Streptococcus agalactiae* and *Escherichia coli*, accounting for two-thirds of all cases, while *Streptococcus pneumoniae* and *Listeria monocytogenes* are less commonly reported [10].

The use of fosfomycin in combination with beta-lactams has been advocated for complicated meningoencephalitis caused by these bacteria [11]. Due to its small size and lack of binding to blood proteins, fosfomycin crosses the blood–rachis barrier; moreover, in the course of meningeal inflammation, its concentration in the cerebrospinal fluid (CSF) increases [12,13].

The 2016 guidelines of the European Society of Clinical Microbiology and Infectious Diseases (ESCMID) assigned a role to fosfomycin only in the treatment of *Staphylococcus aureus* meningitis as an alternative regimen, always featured in combination therapy, regardless of the patient’s age [10].

A literature review published in 2020 by Tsegka et al. on the use of fosfomycin in the CNS reported that fosfomycin is almost exclusively used as combination therapy (87%) at a daily dose ranging from 14 to 24 g/day in the adult population and ranging from 100 to 750 mg/kg/day in the pediatric population. CSF sterilization was achieved in most patients (97.2%) and 93.8% of patients were cured. Since fosfomycin was strictly used in combination, its actual contribution to the success of the therapy could not be accurately defined [14]. According to the literature, fosfomycin is very safe, with electrolyte alteration being the major adverse effect observed [15].

Considering the limited number of cases documented in the literature regarding the use of fosfomycin in neonates, and especially in those with CNS infection, here, we report and describe six patients from a neonatal intensive care unit (NICU) with CNS infection treated with fosfomycin in a combination regimen.

## 2. Results

We identified six neonates (four male and two female) in our search (Table 1).

### 2.1. Case 1

A male neonate was born by cesarean section due to the threat of preterm delivery in a monochorionic and diamniotic twin pregnancy after egg donation. He was an extremely preterm newborn (gestational age of 27 + 2 weeks) with an extremely low birth weight (830 g) and an Apgar score of 1 and 9 at 1 and 5 min after birth, respectively. Immediately after birth, he required non-invasive ventilation and was subsequently admitted to the NICU, where empirical antimicrobial therapy with ampicillin and gentamicin was initiated and fluconazole prophylaxis was started. Treatment was confirmed after the isolation of *Enterocuccos faecalis* and *E. coli* from placental samples and continued for 9 days. The newborn was also treated with indomethacin for patent ductus arteriosus. In the 28 days following birth, progressive clinical stabilization was observed, with the discontinuation of non-invasive ventilation and a gradual reduction in oxygen supplementation.

At 28 days of life, the newborn developed purulent eye secretions; a biocular swab revealed *Serratia marcescens*, leading to topical therapy with tobramycin.

At 36 days of life, there was a sudden decline in general conditions, with significant respiratory distress requiring endotracheal intubation and invasive ventilation. The clinical picture was accompanied by metabolic acidosis and arterial hypotension with oligo-anuria. Blood and urine cultures were performed with negative results. Meropenem and amikacin therapy was initiated. On the fourth day of antibiotic therapy, a trans-fontanelle ultrasound (head ultrasound, HUS) was performed, showing evidence of multiple cerebral abscesses. Given the extremely severe clinical picture and the lack of clinical–laboratory improvement, the ongoing therapy was supplemented with the addition of fosfomycin at a dosage of 100 mg/kg/day. During antibiotic therapy, no adverse effects to treatment were observed. 

**Figure 1:**
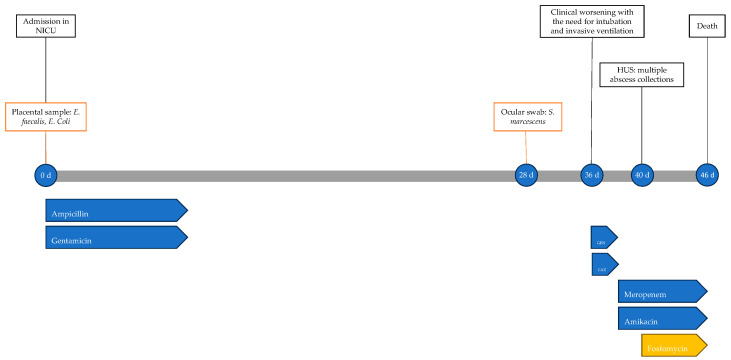
Timeline of infectious events and antibiotic therapy of Case 1. HUS: head ultrasound; CAZ: ceftazidime; GEN: gentamicin.

The newborn’s condition remained gravely stable until 45 days of life, when there was further clinical decline, with worsening metabolic acidosis; he was unresponsive to resuscitation attempts and the neonate died the following day at 46 days of life. The timeline of antimicrobial therapy, microbiological isolations and instrumental data is shown in Figure 1.

### 2.2. Case 2

A male newborn was delivered spontaneously at term (39 weeks + 1 gestational age), with an Apgar score of 9 at 1 min and 10 at 5 min and good adaptation to extrauterine life. He was discharged at 3 days of life with normal growth parameters.

At 21 days of life, he presented to the Emergency Department due to poor appetite and drowsiness for the last 24 h. On evaluation, the neonate appeared tachycardic and febrile, with a complaining cry, and subsequently had a clonic seizure requiring midazolam. Blood tests revealed elevation of C-reactive protein (CRP) levels (90 mg/L, normal value < 5 mg/L). Antibiotic therapy with gentamicin was initiated and the patient was admitted to the NICU, where blood cultures and a lumbar puncture with CSF were performed and ampicillin was added.

Blood cultures and CSF grew *S. agalactiae* and dexamethasone was added to the ongoing antibiotic therapy. The patient was being closely monitored due to depression of cerebral electrical activity. Subsequently, due to the new onset seizure, antiepileptic therapy was initiated. Within the first few hours in the NICU, episodes of apnea with respiratory distress were observed, necessitating endotracheal intubation and invasive ventilation. In the following days, the neurological status improved, with a reduction in seizures, and respiratory function improved, with weaning off invasive ventilation to spontaneous breathing in room air.

At 12 days of antibiotic therapy, brain magnetic resonance imaging (MRI) showed findings compatible with subacute meningoencephalitis with multiple minute focal alterations of deep white matter, numerous minute cavitations, some areas of enhancement, and rare hemosiderin residues. Extra-axial purulent collections along the convexity of both hemispheres, predominantly at the vertex, were observed, without clinically significant compressive effects. Finally, the ventricular spaces appeared diffusely enlarged.

At 15 days of antibiotic therapy, a fever episode recurred with crying, associated with significant elevation of inflammatory markers (CRP 238.4 mg/L); therefore, ampicillin was discontinued and replaced with ceftaroline. Microbiological investigations showed a methicillin-resistant *Staphylococcus epidermidis* (MRSE) from peripheral blood cultures (considered a contaminant). A new lumbar puncture showed low-titer HSV1 (Herpes Simplex Virus 1)-DNA (261 cp/mL), with titers increasing to 21,068 cp/mL of HSV1-DNA on the following day; so, antiviral therapy with acyclovir was started. On the same day, a trans-fontanelle ultrasound showed purulent material in the subarachnoid space, and the current therapy was further intensified by adding metronidazole.

On the 38th day of hospitalization, due to the persistent elevation of inflammatory markers (CRP 153.1 mg/L), the antibiotic therapy was shifted to meropenem and fosfomycin. Acyclovir therapy was discontinued after 21 days, while meropenem and fosfomycin were discontinued after 12 days.

At the end of therapy, the infant showed progressive clinical improvement and remained afebrile, and CRP levels declined significantly (7.1 mg/L at discharge). No adverse effects to the drugs were reported, and in particular, during treatment with fosfomycin, electrolytes were closely monitored, with no evidence of dysionias.

**Figure 2:**
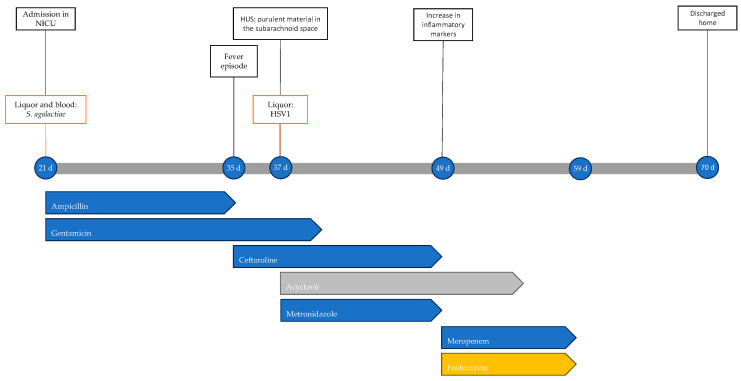
Timeline of infectious events and antibiotic therapy of Case 2. HUS: head ultrasound.

At 70 days of life, the child was discharged in fair general condition. At approximately 3 years from the acute event, he continues antiepileptic therapy with good seizure control, global developmental delay, and microcephaly (head circumference at 2 years of age was 46 cm, below the 2nd percentile). Figure 2 shows the timeline with the most important clinical events, microbiological isolations, instrumental data and antimicrobial therapy performed.

### 2.3. Case 3

A female neonate, born prematurely (26 weeks + 6 gestational age) with a low birth weight (1020 g), discharged from the neonatal pathology ward at 58 days of life in good general condition, was diagnosed with extreme prematurity, hyaline membrane disease, and mild bronchopulmonary dysplasia.

At 65 days of life, the newborn developed respiratory distress, apnea, desaturation, and feeding difficulties. She was brought to the Emergency Department with significant respiratory distress, tachycardia, hypotonia, and hyporeactivity, and was subsequently admitted to the NICU. Blood tests showed leukopenia (white blood cells WBCs, 1910/μL; normal value, 5000–195,000/μL), slight elevation of CRP levels (8.2 mg/L), and elevated procalcitonin (PCT, 8.93 ng/mL; normal value < 0.5 ng/mL). Upon admission to the ward, high-flow ventilation was initiated, and empirical antibiotic therapy with ampicillin, cefotaxime, and amikacin was started pending the result of blood cultures and spinal tap. The CSF chemical–physical analysis showed clear, colorless fluid with elevated protein levels (1180 mg/L; normal value, 150–450 mg/L) and no cells.

**Figure 3:**
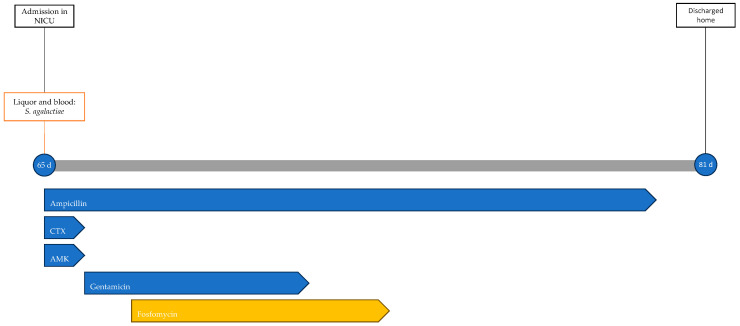
Timeline of infectious events and antibiotic therapy of Case 3. CTX: cefotaxime; AMK: amikacin.

Blood and CSF cultures revealed *S. agalactiae*. Accordingly, the ongoing therapy was optimized to ampicillin and gentamicin. On the second day of hospitalization, the worsening of respiratory dynamics called for endotracheal intubation and invasive mechanical ventilation. Due to the lack of clinical improvement and the persistence of significant inflammation indices (CRP, 210 mg/L; PCT, 90.4 ng/mL), fosfomycin was added to the ongoing therapy on the third day at a dosage of 100 mg/kg per day divided into two administrations. In the following days, gradual clinical improvement was observed, with weaning off ventilation to spontaneous breathing in room air. Gentamicin therapy was discontinued after 5 days and fosfomycin after 6 days, while ampicillin therapy continued for a total of 14 days. No adverse events related to antibiotic therapy were observed during treatment. During hospitalization, the patient underwent EEG (electroencephalogram), revealing abnormalities in the organization of electrical activity without seizures. Additionally, a head ultrasound was performed, showing signs of initial meningeal irritation. An MRI showed increased enhancement only at the level of the III, V, and VII cranial nerves. The patient was discharged home at 81 days of life after 16 days of hospitalization. The patient's treatment timeline is shown in Figure 3.

She continues neonatal, neuropsychiatric, ophthalmological, and audiological follow-up, with normal psychomotor and sensory development. 

### 2.4. Case 4

A male neonate was born at term (38 weeks gestational age) via operative delivery with vacuum extraction due to cardiotocographic abnormalities, with Apgar scores at 1 and 5 min of 9 and 10, respectively, and good adaptation to extrauterine life with normal objective findings. At two days of life, he presented with pallor, hypotonia, tachypnea, and fever. Following microbiological investigations, empirical antibiotic therapy with ampicillin and gentamicin was initiated. Subsequently, due to the worsening of general conditions with seizures and apnea, the neonate was transferred to the NICU, where endotracheal intubation was performed. Blood tests at admission showed leukopenia (WBCs, 3740/μL) and elevated inflammatory markers (CRP, 288.8 mg/L). A lumbar puncture revealed turbid, xanthochromic CSF, with elevated protein levels (4391 mg/dL), low glucose levels (14 mg/dL), and pleocytosis (15,494/mmc, 100% neutrophil granulocytes; normal value < 10/mmc). Prior to obtaining additional culture results, cefotaxime was added to the ongoing therapy, and antiepileptic therapy was initiated.

**Figure 4:**
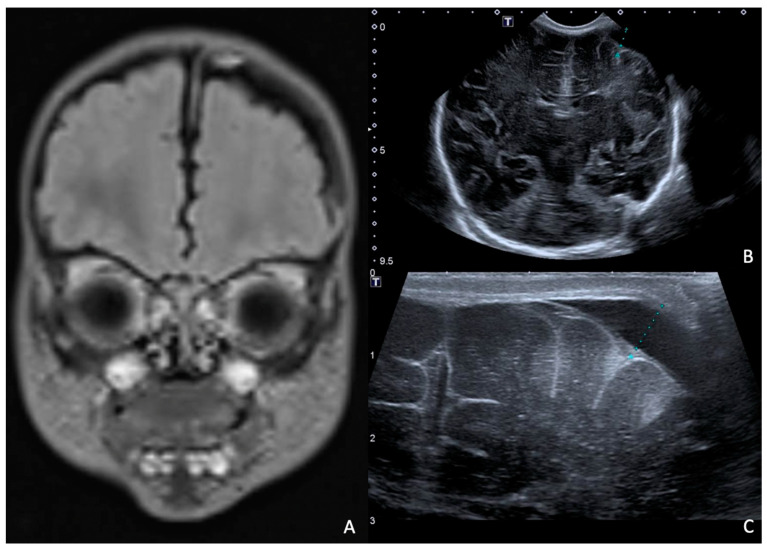
(**A**), Brain MRI in FLAIR sequence with evidence of left parietal subdural collection and multiple diffuse supra- and infratentorial subcentimetric collections. (**B**,**C**) HUS: left parietal subdural collection, with maximum thickness of 7 mm.

Rapid molecular tests on CSF (later confirmed by CSF and blood cultures) revealed a pan-sensitive *E. coli* K1. Ampicillin and gentamicin were discontinued and replaced by fosfomycin, continuing with cefotaxime. At eight days of life, a brain MRI was performed, showing a collection in the left frontal subdural site with a maximum thickness of approximately 7 mm and multiple subcentimetric subtentorial and supratentorial collections compatible with empyema; the collection in the left parietal site was subsequently evaluated by ultrasound until resolution (Figure 4). Therapy was prolonged for a total of 24 days (22 with fosfomycin), with no adverse events reported.

The newborn progressively improved and was extubated, oxygen support was reduced to spontaneous breathing in room air, and hypotonia was resolved. The ophthalmological and audiometric evaluations were normal.

**Figure 5:**
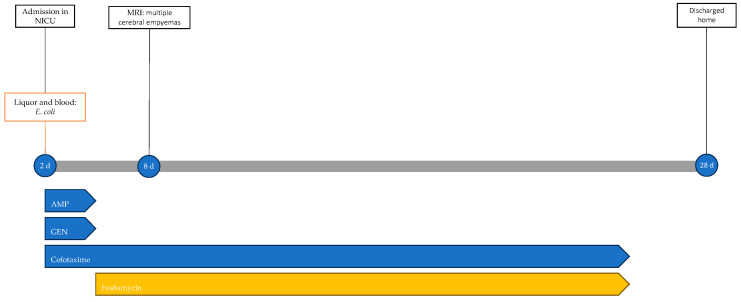
Timeline of infectious events and antibiotic therapy of Case 4. MRI: magnetic resonance imaging; AMP: ampicillin; GEN: gentamicin.

The neonate was discharged home at 28 days of life in good general condition and without sequelae; the resolution of epidural collections was also confirmed by ultrasound. Figure 5 shows the infectious events and antibiotic therapy performed during hospitalisation. 

He underwent follow-up with neonatal and neuropsychiatric care, showing psychomotor development above average for his age.

### 2.5. Case 5

A male neonate, born at home following precipitous delivery at 39 + 3 weeks of gestational age, was subsequently transferred to the hospital, showing good adaptation to extrauterine life, with a 10 min Apgar score of 10. He was discharged at 3 days of life in good general condition.

At 5 days of life, he developed fever and feeding difficulties. He was brought to the Emergency Department, where empirical antibiotic therapy with ampicillin/sulbactam, cefotaxime, gentamicin, and amikacin was initiated, and the newborn was admitted to the NICU. Blood cultures and a lumbar puncture were performed, revealing turbid CSF with low glucose levels (<2 mg/dL), increased protein levels > 6000 mg/dL, and pleocytosis (12,330/μL). Rapid molecular tests and cultures of CSF and blood cultures revealed a pan-sensitive *E. coli* K1. Following microbiological isolation, therapy with amikacin and ampicillin/sulbactam was discontinued and fosfomycin was added to the therapy.

**Figure 6:**
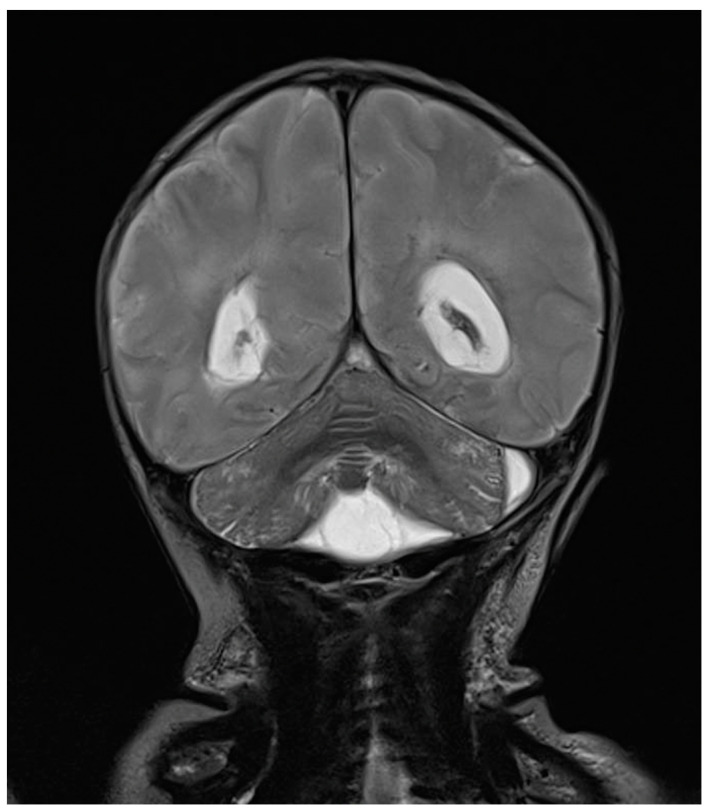
Brain MRI in T2 sequence: picture compatible with meningoencephalitis associated with hypoxic suffering. Infratentorial empyema with small amounts of pus in ventricles supratentorially. Enlargement of lateral ventricles.

**Figure 7:**
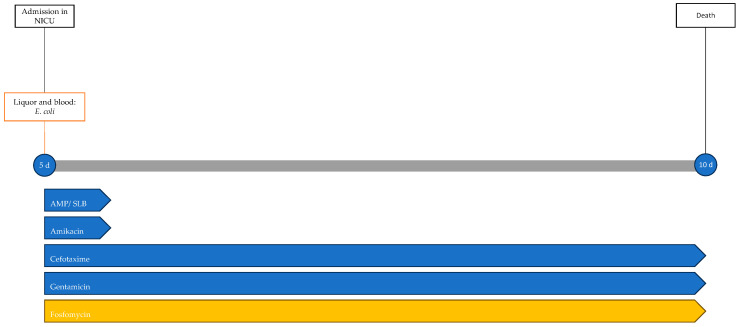
Timeline of infectious events and antibiotic therapy of Case 5. AMP/SLB: ampicillin/sulbactam.

One day after the start of antibiotic therapy, the patient’s condition worsened, with subsequent epileptic seizures and episodes of bradycardia and apnea with desaturation, prompting the initiation of antiepileptic therapy, endotracheal intubation, and invasive mechanical ventilation. In the following days, further deterioration was observed, with progressive cerebral hypoperfusion and ventricular dilation noted on the trans-fontanelle ultrasound. The MRI showed findings consistent with meningoencephalitis with associated hypoxic injury, as well as sub- and supratentorial empyema (Figure 6). The patient was evaluated by neurosurgery without indications for intervention. The neonate died at 10 days of life. The infectious history and antibiotic therapy are schematized in the timeline in Figure 7.

### 2.6. Case 6

A female neonate was born late preterm (36 + 4 weeks of gestational age) via emergency cesarean section due to fetal bradycardia. The pregnancy was characterized by maternal anemia requiring transfusion, a threat of preterm delivery at 33 + 6 weeks, and polyhydramnios. At birth, the newborn was cyanotic, hypotonic, and in asystole. Her Apgar score at 1 and 5 min was 0 and 1, respectively. The newborn underwent resuscitation, with gradual recovery of the heart rate that normalized by 13 min of life despite severe hypoxia. Ampicillin, gentamicin, and antiepileptic therapy were initiated, and the newborn was transferred to the NICU. 

Inotropic support with dobutamine and inhaled nitric oxide was initiated due to signs of post-capillary pulmonary hypertension. The trans-fontanelle ultrasound findings were consistent with hypoxic–ischemic encephalopathy. Further findings from a chest X-ray were indicative of early-onset pneumonia, so antibiotic therapy was modified, replacing ampicillin with ampicillin/sulbactam.

**Figure 8:**
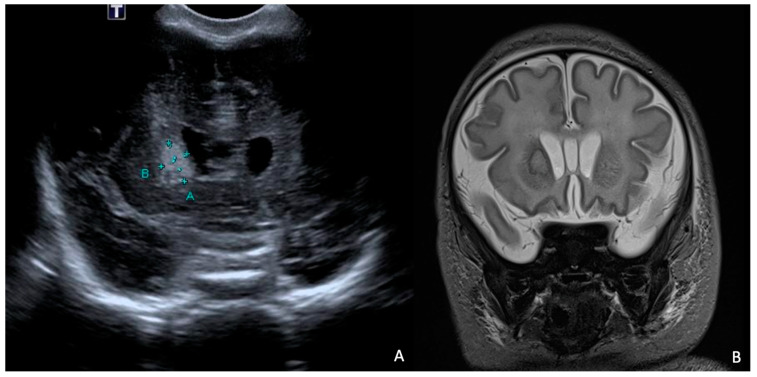
HUS (**A**) and brain MRI in T2 sequence (**B**): abscess of approximately 1 cm in diameter (delimited by the letters A and B in (**A**)) at level of right caudate nucleus in context compatible with hypoxic–ischemic encephalopathy. MRI in T2 sequence showed evidence of rim and hyperintense core.

At 7 days of life, an HUS and a brain MRI were performed, showing a cerebral abscess lesion in the right caudate nucleus (Figure 8). Biochemical parameters in the CSF were normal. Blood and CSF cultures as well as PCR for neurotropic viruses and bacteria were negative. Empirical antibiotic therapy was modified by discontinuing ampicillin/sulbactam and starting broad-spectrum therapy with ampicillin, cefotaxime, and metronidazole.

Inotropic therapy was discontinued by day 8 of life, and oxygen supplementation was terminated by day 16 of life. 

At 14 days of life, a second trans-fontanelle ultrasound was performed, showing substantial stability of the abscess lesion. Therefore, the ongoing antibiotic therapy was discontinued, and meropenem was initiated. At 21 days, after another unchanged ultrasound control, fosfomycin was also added.

**Figure 9:**
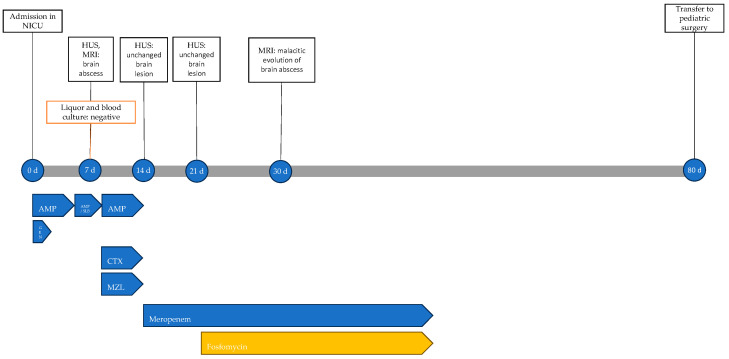
Timeline of infectious events and antibiotic therapy of Case 6. HUS: head ultrasound; MRI: resonance magnetic imaging; AMP: ampicillin; AMP/SLB: ampicillin/sulbactam; GEN: gentamicin; CTX: cefotaxime; MZL: metronidazole.

At 30 days of life, a repeat MRI showed malacic evolution of the known abscess lesion and treatment was extended to a total of 6 weeks. The patient showed no adverse events related to antibiotic therapy. The infectious history with microbiological and instrumental investigations performed and the antibiotic therapy carried out is shown in Figure 9.

Slow neurological improvement was observed, critical episodes ceased, and antiepileptic therapy was gradually discontinued. 

Due to poor feeding with frequent vomiting and regurgitation episodes, Nissen fundoplication and laparoscopic gastrostomy were performed at 80 days of life.

The newborn was discharged at days 155 of life with hypotonia of the axis, psychomotor delay, and visual and spontaneous motor function abnormalities with hyperexcitability.

## 3. Discussion

To our knowledge, there are no reported cases in the English literature regarding the use of fosfomycin in newborn CNS infections, with the sole exception of a patient with a brain abscess caused by *Cytrobacter koseri* [16].

Among our four patients with meningitis, two (one with *S. agalactiae* meningitis and one with *E. coli* meningitis) showed complete clinical resolution without sequelae. One newborn, without other comorbidities at birth, died due to *E. coli* meningitis and one patient with *S. agalactiae* infection developed subcortical encephalomalacia with psychomotor deficits and epilepsy. It is noteworthy to report that it cannot be ultimately determined whether these sequelae were directly attributable to *S. agalactiae* meningitis or to the subsequent HSV1 meningoencephalitis. 

Bacterial meningitis is the most common infection of the CNS at any age; based on the 2016 Global Burden of Disease report, bacterial meningitis accounts for 320,000 fatalities per year, placing it among the top 10 causes of death attributed to communicable diseases [17]. According to international guidelines, we used empirical therapy for neonatal meningitis with combination regimens containing ampicillin/amoxicillin, cefotaxime, and an aminoglycoside [18]. In the four cases presented here, these regimens were later replaced by a fosfomycin-containing regimen due to the worsening of clinical and laboratory parameters. Fosfomycin was chosen for salvage therapy due to its favorable concentration into the CNS.

Regarding our two patients with cerebral abscesses, the first might have had local dissemination from *S. marcescens* conjunctivitis, while the second developed an abscess in an area injured by severe hypoxic episodes. Both cases had conditions (extremely low birth weight, extreme prematurity, and patent ductus arteriosus or ischemic injury) that justify the severe course of infection; one of them died and the other survived with significant sequelae from a neonatal hypoxic–ischemic event. The treatment of cerebral abscesses would typically involve a combination of prolonged antibiotic therapy and surgical drainage [19]; however, our patients were exclusively treated with antibiotic therapy. In both cases, fosfomycin therapy was introduced as a secondary measure after clinical–laboratory deterioration.

The other single case reported in the literature describes a newborn who underwent surgical drainage and received targeted antibiotic therapy for 6 weeks with meropenem and fosfomycin. The patient showed resolution of the infectious process, resulting in mild impairment of gross motor skills [16]. Several factors may have contributed to his better outcome compared to our cases. Firstly, this neonate had no comorbidities, and was born at term and with a healthy weight. Additionally, surgical drainage likely played a significant role in the successful resolution of the process.

Cerebral abscesses are generally rare in the pediatric population but remain associated with significant mortality and long-term sequelae. With improvements in diagnostic procedures, surgical techniques, and the optimization of antibiotic therapy, current mortality rates have reduced to 10%. Predisposing factors include congenital heart anomalies and contiguous infections, with transmission occurring through contiguity or hematogenously [20].

All our patients were treated with different therapeutic regimens in terms of combinations and the duration of therapy (from 5 to 28 days); the dosage of fosfomycin was as indicated in the product label, namely 200 mg/kg per day divided into three administrations in full-term newborns, and 100 mg/kg per day divided into two administrations in preterm newborns with a gestational age plus postnatal age less than 40 weeks.

The well-documented side effects of fosfomycin in adults include heart failure and hypokalemia, linked to the sodium load of intravenous fosfomycin, but have not been reported in the pediatric population [15]. The randomized study by Obiero et al. aimed to comparatively assess potential changes in plasma sodium levels during intravenous fosfomycin therapy. The results showed no differences in plasma sodium levels between the two arms. Among the remaining adverse events observed, none were correlated with the study drug, confirming the favorable safety profile of fosfomycin [21]. Our observation confirms these findings.

## 4. Materials and Methods

We searched for and described all neonates with CNS infections who received fosfomycin in the NICU of Spedali Civili of Brescia over a period of 5 years (from June 2018 to June 2023).

For each neonate, demographic, clinical, and microbiological variables and therapeutic strategies are described. In particular, the presence of prematurity, comorbidities, microbiological isolations, a history of antibiotic treatments, and concurrent use of fosfomycin have been evaluated, along with the overall outcome.

## 5. Conclusions

Fosfomycin can be an important alternative option in combating challenging infections of the CNS thanks to its ability to penetrate the blood–brain barrier effectively, its unique mechanism of action, and its synergies with other antibiotic molecules.

Our case series confirms the favorable safety profile of fosfomycin in the neonatal population but is limited; further studies are needed to better explore the role of the molecule in CNS infections, especially in the neonatal context.

## Figures and Tables

**Table 1:** Summary of patients treated with fosfomycin-containing regimens from June 2018 to June 2023. M: male, F: female, GA: gestation age, CSF: cerebrospinal fluid, ADR: adverse drug reaction, HSV1: Herpes virus simplex 1, LGA: large for gestational age, HUS: head ultrasound, MRI: magnetic resonance imaging, EEG: electroencephalogram, AMK: amikacin, AMP: ampicillin, SLB: sulbactam, CTX: cefotaxime, CPT: ceftaroline, CAZ: ceftazidime, GEN: gentamicin, MEM: meropenem, and MZL: metronidazole.

	0 Days, M	21 Days, M	64 Days, F	2 Days, M	5 Days, M	0 Days, F
Diagnosis	Brain abscesses	Meningitis with multiple subdural empyemas	Meningitis	Meningitis with multiple subdural empyemas	Meningitis with ventriculitis and subtentoral empyema	Brain abscess
GA	27 + 2 weeks	39 + 1 weeks	26 + 6 weeks	38 weeks	39 + 3 weeks	36 + 4 weeks
Birth weight	830 g	3038 g	1020 g	2730 g	3664 g	3930 g
Comorbidities	Patency of the Botallo’s duct	None	Membrane diseases hyaline, bronchopulmonary dysplasia	None	None	Hypoxic–ischemic encephalopathy, LGA, gastro-esophageal reflux, pulmonary hypertension
Clinical presentation	Apnea, respiratory failure, metabolic acidosis	Poor feeding, drowsiness, fever, tachycardia, plaintive crying, hypotonia, seizures, respiratory failure	Respiratory distress and failure, poor feeding, hypotonic, hyporeactive	Pallor, hypotonia, polypnea, respiratory failure, fever, seizure	Fever, plaintive crying, irritability, polypnea, respiratory failure, seizure	Multiple organ failure, coma, seizure
CSF	None	*S. agalactiae* HSV1	*S. agalactiae*	*E. coli* K1	*E. coli* K1	None
Other significant sample	Ocular, pharyngeal, rectal swabs, gastric juice	Blood	Blood, nasopharyngeal aspirate, pharyngeal, nasal, and rectal swabs	Blood, pharyngeal, ear, and nasal swabs, gastric juice, meconium	Blood, urine, nasopharyngeal aspirate, pharyngeal, ocular, and nasal swabs, meconium	None
Imaging	HUS	HUS, encephalon and brainstem MRI, EEG	HUS, encephalon and brainstem MRI, EEG	HUS, encephalon and brainstem MRI, EEG	HUS, encephalon and brainstem MRI, EEG	HUS, encephalon and brainstem MRI, EEG
Previous antibiotics	CAZ, GEN, AMP	AMP, GEN, CPT, MZL	AMK, CTX	AMP	AMP/SLB	CTX, AMP, MZL
Concomitant antibiotics	MEM, AMK	MEM	AMP, GEN	CTX	CTX, AMK	MEM
Days of fosfomycin	7	12	5	21	5	28
Dosage	100 mg/kg/day q12h	200 mg/kg/day q8h	100 mg/kg/day q12h	200 mg/kg/day q8h	200 mg/kg/day q8h	200 mg/kg/day q8h
ADR	None	None	None	None	None	None
Outcome	Death	Cortico-subcortical encephalomalacia with psychomotor delay and focal epilepsy	None	None	Death	Axis hypotonia and psychomotor delay

## Data Availability

No new data were created or analyzed in this study. Data sharing is not applicable to this article.
